# Development of HPLC Method for Catechins and Related Compounds Determination and Standardization in Miang (Traditional Lanna Fermented Tea Leaf in Northern Thailand)

**DOI:** 10.3390/molecules26196052

**Published:** 2021-10-06

**Authors:** Sunanta Wangkarn, Kate Grudpan, Chartchai Khanongnuch, Thanawat Pattananandecha, Sutasinee Apichai, Chalermpong Saenjum

**Affiliations:** 1 Department of Chemistry, Faculty of Science, Chiang Mai University, Chiang Mai 50200, Thailand; sunanta.w@cmu.ac.th (S.W.); kgrudpan@gmail.com (K.G.); 2 Center of Excellence for Innovation in Analytical Science and Technology (I-ANALY-S-T), Chiang Mai University, Chiang Mai 50200, Thailand; thanawat.pdecha@gmail.com (T.P.); sutasinee.apichai@gmail.com (S.A.); 3 Cluster of Excellence on Biodiversity-based Economic and Society (B.BES-CMU), Chiang Mai University, Chiang Mai 50200, Thailand; ck_biot@yahoo.com; 4 Research Center for Multidisciplinary Approaches to Miang, Chiang Mai University, Chiang Mai 50200, Thailand; 5 Division of Biotechnology, Faculty of Agro-Industry, Chiang Mai University, Chiang Mai 50200, Thailand; 6 Department of Pharmaceutical Sciences, Faculty of Pharmacy, Chiang Mai University, Chiang Mai 50200, Thailand

**Keywords:** HPLC, method validation, *Miang*, catechins, caffeine, gallic acid

## Abstract

High performance liquid chromatography (HPLC) for catechins and related compounds in *Miang* (traditional Lanna fermented tea leaf) was developed to overcome the matrices during the fermentation process. We investigated a variety of columns and elution conditions to determine seven catechins, namely (+)-catechin, (−)-gallocatechin, (−)-epigallocatechin, (−)-epicatechin, (−)-epigallocatechin gallate, (−)-gallocatechin gallate, (−)-epicatechin gallate, as well as gallic acid and caffeine, resulting in the development of reproducible systems for analyses that overcome sample matrices. Among the three reversed-phase columns, column C (deactivated, with extra dense bonding, double endcapped monomeric C18, high-purity silica at 3.0 mm × 250 mm and a 5 µm particle size) significantly improved the separation between *Miang* catechins in the presence of acid in the mobile phase within a shorter analysis time. The validation method showed effective linearity, precision, accuracy, and limits of detection and quantitation. The validated system was adequate for the qualitative and quantitative measurement of seven active catechins, including gallic acid and caffeine in *Miang,* during the fermentation process and standardization of *Miang* extracts. The latter contain catechins and related compounds that are further developed into natural active pharmaceutical ingredients (natural APIs) for cosmeceutical and nutraceutical products.

## 1. Introduction

Tea (*Camellia sinensis*, family Theaceae) is the most frequently consumed beverage worldwide and a rich natural source of polyphenols, flavonoids, and alkaloids. Numerous studies have identified the characteristic constituents of tea leaves into two main groups: catechins and alkaloids. The active catechins are (+)-catechin (C), (−)-epicatechin (EC), (−)-gallocatechin (GC), (−)-epigallocatechin (EGC), (−)-catechin gallate (CG), (−)-gallocatechin gallate (GCG), (−)-epicatechin gallate (ECG), and (−)-epigallocatechin gallate (EGCG), whereas the major active alkaloid is caffeine (Caf.). EGCG is the major component of unfermented green tea, accounting for approximately 10–50% of tea catechins overall [1]. In fermented green tea, the major component is GC, with less EGCG content [2]. One of the most intriguing properties of tea catechins is protection against cancer, diabetes, hypertension, dyslipidemia, and cardiovascular diseases [3,4,5,6]. Several studies have reported that the antioxidant activity is higher in green tea due to higher amounts of EGCG and EGC [7,8,9]. In addition, tea contains caffeine, which stimulates the central nervous system and induces short-term increases in blood pressure [10].

In Northern Thailand, two varieties of tea are cultivated, namely Assam tea (*Camellia sinensis* var. *assamica*) and Chinese tea (*Camellia sinensis* var. *sinensis*). Assam tea leaves are larger than those of the Chinese variety. In 2007, 77% of fresh tea leaves produced in Thailand were processed into dried tea, and 23% produced *Miang* (traditional Lanna fermented tea leaf) [11]. In Northern Thailand, every generation has inherited *Miang* production, which is known as a typical fermented tea produced from the Assam variety. After harvesting fresh tea leaves, the tea leaves are steamed, bunched, and fermented via endo-oxidation from 13 days to 4 months [12]. The color of *Miang* ranges from yellow-green to dark green. According to local wisdom, traditional *Miang* production is categorized into two processes: the filamentous fungi growth-based process or two-step fermentation process and the non-filamentous fungi-based fermentation process [13]. *Miang*’s taste ranges from tart to sour. As a caffeine source, *Miang* is often eaten as a snack during the workday to increase alertness. As a unique product exclusive to the northern provinces of Thailand, it is also used in local ceremonial events, i.e., Chiang Rai, Chiang Mai, Nan, Lampang, Phare, Phayao, and Mae Hong Son. However, some are exported to Laos, Myanmar, and Southern China [13,14]. *Miang* contains high amounts of bioactive compounds, including EC, C, GC, EGCG, ECG, and EGC [15]. During the fermentation period, the phytochemicals and nutritional compounds of steamed tea leaves are used by enzymes derived from various micro-organisms linked to catalytic biotransformation processes that produce metabolites, including polyphenolic compounds, organic acids, amino acids, and health-related bioactive metabolites [16,17,18]. *Miang* plays a key role as a natural anti-oxidative agent in the body; therefore, consuming *Miang* has many health benefits. Owing to increasing interest in the quality of *Miang* products, there is a strong demand for efficient quality control measures to ensure the proper content of active catechins and related compounds.

Extensive studies on determining catechins and caffeine in several types of tea have been reported using high-performance liquid chromatography (HPLC) and capillary electrophoresis (CE) [19,20,21,22,23,24,25,26,27]. Although these methods demonstrate the separation and detection of tea catechins, they have notable limitations regarding sample matrices and their complexity. For example, Kanpiengjai et al. [14] and Chaikaew et al. [28] reported that tannin-tolerant lactic acid bacteria and tannin-tolerant yeasts produce health-benefiting compounds, including phenolic compounds, organic acids, and volatile acids during the fermentation process. These compounds become the interfere matrix. Therefore, a new method must be developed to overcome them. Furthermore, few data have been reported on determining individual catechin and caffeine in *Miang*. Individual catechin amounts in *Miang* tea were reported by Sirisa-Ard et al. [15]. The amount of catechin and catechin derivatives was analyzed by HPLC equipped with a UV detector for wavelengths between 280 and 210 nm. A reversed-phase C18 column (4.6 mm × 250 mm; Waters, Ireland) with a column temperature between 25 and 30 °C was used. The linear gradient of elution was followed by 0–100% of mobile phase A (86% *v*/*v* phosphoric acid (0.2% *v*/*v*) in 12% acetonitrile and 1.5% *v*/*v* tetrahydrofuran) for 30 min and gradually increased mobile phase B (73.5% *v*/*v* phosphoric acid (0.2% *v*/*v*) in 25% acetonitrile and 1.5% *v*/*v* tetrahydrofuran) from 0–100% for 10 min and holding for 20 min with a flow rate of 1 mL/min. The results showed that GC, EGC, C, EC, EGCG, and ECG amounts in *Miang* tea (B02D) were 9.65, 0.84, 16.13, 61.60, 6.46, and 1.93 mg/g in dry samples, respectively. The number of active ingredients in *Miang* was also determined using the HPLC method [29,30]. However, long-term analysis was required in the previous reports because the optimum condition lacked investigation. There have been no reports of using HPLC to separate and detect individual catechins, GA, and Caf.to simultaneously overcome the matrix interference of the compounds during Assam tea fermentation (*Miang*). Small amounts of catechin (1.34–8.71 mg/g) were found in all *Miang* samples, whereas relatively high EGCG contents (range from 18.50 to 37.24 mg/g) varied among treatments with total phenolic compounds at around 26.24–48.76 mg/g [26].

The total phenolic content, flavonoid content, proanthocyanidin content, and antioxidant activities were reported for three different maturities of *Camellia sinensis* var. *assamica* leaves from Northern Thailand using various extracting solvents. The results revealed that the highest yields were for shoot tea with hot DI water extraction [27]. Sampanvejsobha et al. reported that the amount of total catechins (0.767–3.543% dry weight), caffeine (0.747–1.428% dry weight), tannins (0.963–1.831% dry weight), theanine (1.993–3.686% dry weight), and other ions in astringent *Miang* collected from markets in Chiang Mai, Chiang Rai, and the Phare provinces [12]. Huang et al. [31] studied the microbial transformation of traditional pickled tea fermented under anaerobic conditions. Based on the analysis of changes in the chemical components and sensory quality of pickled tea, properly controlling the fermentation time is a key step for obtaining the desired quality. After 7 days of submerged fermentation, the pickled tea improved sensory quality, and its taste was less bitter and astringent [31].

In this study, we developed an HPLC system for catechins and related compounds -determination and standardization in *Miang* extracts. The study involved comparing three HPLC columns for separating catechin, their derivatives, GA, and Caf. in *Miang*. To obtain accurate data and an efficient HPLC routine method, our research concerning *Miang* analyzed the linearity, accuracy, precision, limit of detection (LOD), and limit of quantitation (LOQ) of the validated HPLC method.

## 2. Results and Discussion

### 2.1. Comparative Separation of Columns

**Figure 1:**
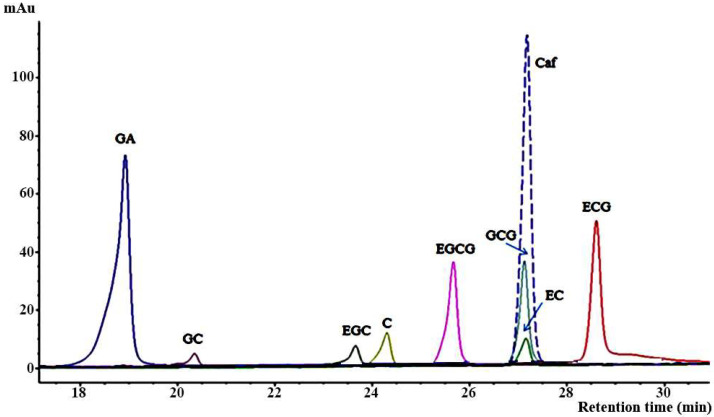
Chromatographic separation of seven catechins containing gallic acid and caffeine in a standard mixture using column A under a suitable gradient elution (see Table 1).

The comparative separation of columns was performed according to the various matrices produced during the biotransformation of *Miang*. The study began with an attempt to reproduce several separations of catechins, GA, and Caf. using three HPLC columns for determining and standardizing the amount of compounds of interest in *Miang* samples. Chromatographic conditions using three columns of C18 were optimized for specificity, resolution, and analysis time at room temperature. Methanol or acetonitrile mixed with either phosphoric acid or acetic acid were studied for their use in mobile phases. The conditions of each column were modified from previous works. Oboh et al. used column A to separate catechin, GA, and Caf., but must be modified to separate the individual catechins [32]. Under various gradient elution systems, the initial effort was performed on column A (deactivated, non-endcapped monomeric C18 column and silica purity were not provided). As shown in Figure 1, six compounds of interest were separated completely within 29 min, and the gradient elution shown in Table 1 GCG, EC, and Caf. were coeluted. Additionally, ECG showed significant peak tailing; according to a previous study, this was likely caused by unfavorable interaction of the basic compound with accessible acidic silanols [23]. An occurring peak fronting in some compounds including GA, GC, EGC, and C may cause by the concentrations or volume injected were overloading of column.

**Figure 2:**
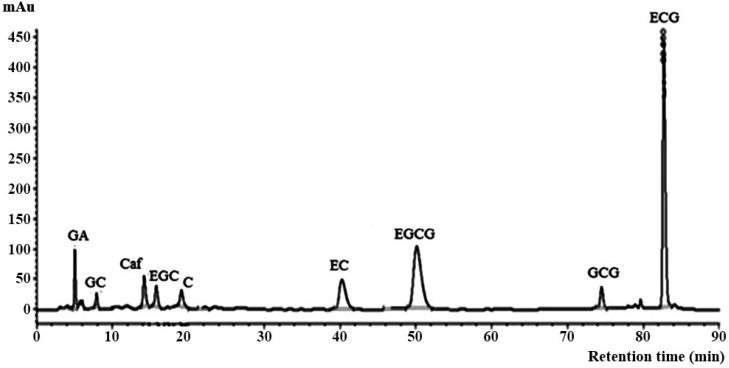
Chromatographic separation of seven catechins containing gallic acid and caffeine in a standard mixture using column B under a suitable isocratic elution (see Table 1).

Column B (deactivated, endcapped monomeric C18, high-purity silica) was tested using methanol in the presence and absence of ethyl acetate mixed with either phosphoric acid or acetic acid as mobile phases modified from a previous report [33]. The separation was performed with ethyl acetate added to the mobile phase gave sharper peak shapes (C, EGCG, and GCG) than those with ethyl acetate absent [34,35,36]. Furthermore, acetic acid has the same effect on the separation, but is not as effective as phosphoric acid. The amount of phosphoric acid in the mobile phase was used in the range of 0.05–0.10% to improve the peak shapes of GA, EGCG, and GCG. Therefore, the separation of seven catechins (GC, EGC, C, EC, EGCG, GCG, and ECG), GA, and Caf. was achieved within 84 min under suitable isocratic condition, as shown in Figure 2 and Table 1.

**Figure 3:**
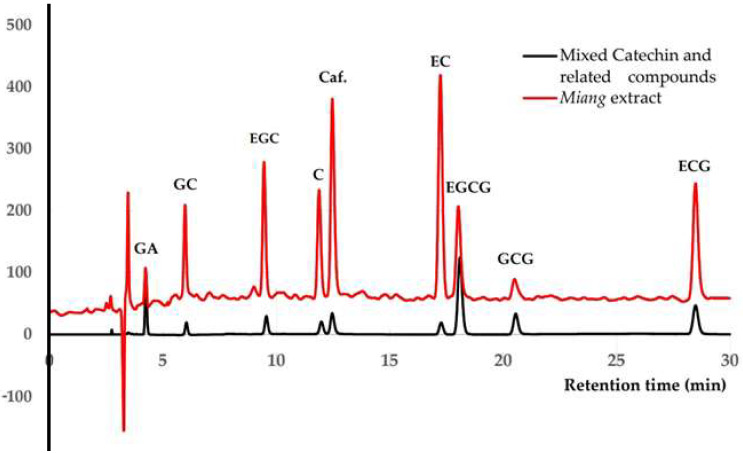
Chromatographic separation of seven catechins containing gallic acid and caffeine in a standard mixture using column C under a suitable gradient elution (see Table 1) comparable to the *Miang* extract.

**Figure 4:**
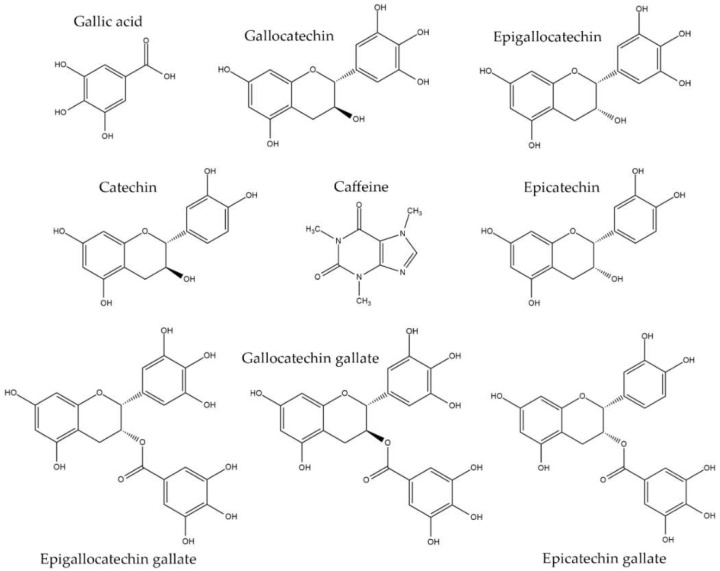
Structure of gallic acid, caffeine, catechin and derivatives.

In most published studies, the mobile phase containing water, acids (trifluoroacetic acid, phosphoric acid, and acetic acid), and either methanol or acetonitrile has been used for catechin analysis of green tea and dried tea leaves [7,22,23,34,35]. Column C and the mobile phase, which comprised a mixture of an eluent A (acetonitrile + 0.1% acetic acid) and B (0.1% acetic acid in water), were employed under various gradient elution systems to shorten the analysis time and improve separation between the seven catechins, GA, and Caf. Among nine compounds of interest, the separation between catechin and caffeine was poor (Rs < 1.0) under the gradient elution system illustrated in Table 1. Adding the volume fraction of methanol into the eluent A (MeOH-ACN mixture) was varied from 0% to 100% in 10% increments. The volume ratio 90:10 MeOH-ACN for eluent A was the only combination that resulted in the separation of all nine compounds within 30 min, as shown in Figure 3 and Table 1. According to the principle of separation in reversed-phase chromatography, the obtained separate order was GA, GC, EGC, C, Caf., EC, EGCG, GCG, and ECG. Regarding the log P-value, logarithms of the partition coefficient are used between solute concentrations in immiscible binary phase solvents, namely water and octanol, which measure the lipophilicity or hydrophobicity of each compound. The hydrophobic compounds, observed from the high log P-values, were distributed into then stationary phase and eluted slowly. By contrast, the hydrophilic compounds, observed from the low values of log P, distributed efficiently into the mobile phase, resulting in quick processing. The log P-values of GA, GC, EGC, C, Caf., EC, EGCG, GCG, and ECG were 1.13, 1.49, 1.49, 1.80, −0.55, 1.80, 3.08, 3.08, and 3.88, respectively, as shown in Table 2, which correspond to the sequence in this study [37]. Moreover, the functional and size of the molecules shown in Figure 4 are involved causing the separation sequence as illustrated in Figure 3. A resolution >1.0 was achieved for all neighboring peaks; this is considered acceptable for analytical purposes as it indicates a 98% separation between two neighboring peaks. Although the baseline shifted due to the change in the mobile phase, it did not affect the detection of individual peaks. The detection wavelength was selected at 210 nm because nearly all compounds of interest exhibited maximum absorbance compared with 230 and 270 nm [7,22,23].

Among the three columns, column B and column C are used in high-purity and inert silica support, further deactivating the C18 chains through endcapping. Our results demonstrated that both columns separated all nine compounds under suitable conditions. In addition to double end-capping via the extra dense bonding of column C, these columns provided a higher quality chromatography than the tested columns.

### 2.2. Method Validation

As illustrated in Figure 2 and Figure 3, both columns separated all nine compounds successfully. As shown in Table 2, some analytical parameters such as linearity, limit of detection (LOD), limit of quantitation (LOQ), and precision were examined to evaluate the method’s performance. The calibration curves of columns C and B were linear in the ranges between 2–20 and 5–35 mg/L, showing correlation coefficients (R^2^) of more than 0.9993 and 0.9970 for each compound, respectively. LOD and LOQ were determined as 3 and 10 standard deviations from the blank signal (*n* = 7), respectively. The values of LOD and LOQ ranged between 0.23–0.68 and 0.67–2.18 mg/L using column C, between 0.10–2.45 mg/L and 0.23–8.19 mg/L using column B, indicating this method’s sufficient sensitivity.

For column C, intraday and interday precisions of retention time expressed as RSD were less than 2%, whereas precisions using column B were less than 3.0%. Considering the peak area, the RSD values of both precisions for all nine compounds using column C were less than 2%. The RSD values of intraday precision for column B were less than 2%, whereas interday precision was less than 4%.

As shown in Table 2, the performance of column C for the separation of all target compounds was superior to that of column B. The method’s accuracy was determined by investigating recovery studies of nine compounds using column C. Assays were performed on three *Miang* extracts in three replicates at each concentration. The recovery of spiked *Miang* extract, in terms of method accuracy, was within the range of 85–106%, and RSD values were less than 8%, as shown in Table 3. The result was satisfactory for the intended purpose and adequate for routine analysis.

### 2.3. Quantitative Analysis in Miang Extracts

In *Miang* extract, sample matrices may cause a bias by increasing or decreasing the peak signal attributed to the measurement. Various extracts of *Miang* samples were analyzed by the validated method with column C to confirm the method’s suitability in determining and standardizing catechins and related compounds. The resulting chromatogram in Figure 3 was compared to a mixed standard. The contents of individual compounds in *Miang* extracts are shown in Table 4. A total of 75% ethanolic solvent exhibited the highest extractability of total catechins (at 60 °C for C, 70 °C for EGC and EGCG, and 80 °C for EC, GC, GCG, and ECG whereas 50% ethanolic solvent showed the highest extractability for GA at 80 °C, and Caf. at 60 °C. Note that the individual catechins, GA, and Caf. were stable at the obtained optimal extraction temperature below 90 °C, as confirmed from the results of previous studies [38]. Previous extraction kinetic studies found that some of the compound contents, including EGC, EC, and Caf., decrease when the extraction temperature increases to 90 °C. The results correspond with those of Liang et al. [39], who reported that 75% ethanol is the highest extractability of total catechins for fresh tea leaves. The individual catechin contents obtained from this optimal extraction, including EGC, C, ECG and EGCG, were higher than previous studies. Nonetheless, only GC and ECG showed lower extractability [15].

## 3. Materials and Methods

### 3.1. Chemicals and Reagents

Standards for catechins and related compounds were used in this work. Catechins and other related compounds were purchased from Sigma-Aldrich (St Louis, Missouri, MO, USA): (+)-catechin (C), (−)-gallocatechin (GC), (−)-epigallocatechin (EGC), (−)-epicatechin (EC), (−)-epigallocatechin gallate (EGCG), (−)-gallocatechin gallate (GCG), (−)-epicatechin gallate (ECG), gallic acid (GA), and caffeine (Caf.). HPLC-grade acetonitrile and methanol, including ethyl acetate, were supplied by Merck (Darmstadt, Germany). Analytical-grade acetic acid (Sigma-Aldrich) and orthophosphoric acid (BDH, Poole, U.K.) were also purchased. HPLC-grade water (18 MΩ) was prepared using a Millipore Milli-Q purification system (Millipore Corp. Bedford, MA, USA) and used to prepare all solutions.

### 3.2. Instrumentation

An HP 1200 series liquid chromatography system (Agilent Technologies, Santa Clara, CA, USA) comprising a vacuum degasser, quaternary pump, auto-sampler, thermostated column compartment, and diode array detector was used. The three reversed-phase LC columns used were column A (4.6 mm × 250 mm, 5 µm particle size; Vertical Chromatography Co., Ltd., Nonthaburi, Thailand), column B (3.0 mm × 250 mm, 5 µm particle size, Wako Pure Chemical Industries, Ltd., Japan), and column C (3.0 mm × 150 mm, 5 µm particle size, Agilent Technologies, Santa Clara, CA, USA) and all columns were equipped with a specific C18 guard column. Isocratic and gradient elution systems were developed using different mobile phases to separate seven tea catechins, GA, and Caf. at flow rates of 0.45, 0.50, and 1.0 mL/min. The detection of analytes was performed by UV detection at 210 and 270 nm.

### 3.3. Sample Extraction

The *Miang* samples were produced by a non-filamentous fungi-based fermentation process and collected from Chiang Dao district, Chiang Mai, Thailand, in October 2018. Prior to the HPLC analysis, they were extracted by three different solvents including 75% ethanol (S1), deionized water (S2), and 50% ethanol (S3) at 60, 70, and 80 °C (T1, T2, and T3), respectively) for 1 h. The duration of each extraction was chosen according to previous studies [38,39] that reported the highest efficiency of all compound extractions at 40 and 10 min and stability at 80 and 65 min by water and ethanol solvent. Then, the extracted solution was evaporated under reduced pressure and dried with a vacuum dryer. Subsequently, the *Miang* extracts dissolved and were filtered through a 0.45 μm membrane filter and 10–80 µL of extracts were analyzed directly by HPLC under suitable conditions. Each extract was analyzed for individual catechins, GA, and Caf. content in three replicates.

### 3.4. Method Validation

Our method was validated according to EURACHEM guidelines [40]. At the concentration corresponding to the middle of the calibration range; the standard mixture of nine compounds was injected with ten replicates for the suitability of the system’s test measurements. The intraday precision (repeatability) and interday precision (within laboratory reproducibility, measurements were performed on three different days) were monitored. We determined the following validation parameters: range, linearity, limit of detection (LOD) and quantitation (LOQ), and accuracy. Linearity was assessed using mixed standard solutions at five concentration levels of each compound. The selected *Miang* extracts were spiked with a mixed standard solution at 5 and 10 times the LOQ used for determining the method accuracy, or relative spiked recovery.

## 4. Conclusions

We found that the monomeric C18 column is preferable to non-endcapped and non-deactivated columns due to their complexity and sample matrices; the qualitative and quantitative analysis of catechins and related compounds in *Miang* samples were successful using endcapped and deactivated columns. Moreover, the presence of acid in the mobile phase is essential for complete separation, especially for GA and GC. The mobile phase was column-dependent in the presence and absence of ethyl acetate for catechin analysis. The proposed HPLC method using column C (3.0 mm × 150 mm, 5 µm particle size) allowed for an accurate quantitation of catechins, GA, and Caf. in *Miang* extracts without interference from other components and performed a single separation in 30 min. The method we developed provides a shorter analysis time compared with previous methods, and effectively overcomes the interference of other metric compounds in *Miang*. Therefore, our method serves as an important reference for the quality control and standardization of *Miang* production, especially since the amounts of active compounds in *Miang* are prone to variation from environmental factors and manufacturing conditions.

## Figures and Tables

**Table 1:** The HPLC conditions of three columns used for separating catechins and related compounds.

Column	Condition		
(1) Column A Deactivated, non-endcapped monomeric C18 4.6 mm × 250 mm, 5 µm particle size with no silica purity provided		MeOH: 0.05% HOAc in H_2_O	
	Mobile phase Gradient elution	Time (min) % of MeOH	
		0	3
		1	3
		21	50
		26	55
		40	95
	Wavelength	270 nm	
	Flow rate	0.5 mL/min	
(2) Column B Deactivated, endcapped monomeric C18, High-purity silica, 3.0 mm × 250 mm, 5 µm particle size and a 2 µm filter attached to both ends of column	Mobile phase Isocratic elution	A: 1% ethyl acetate in MeOH B: 0.1% H_3_PO_4_ in H_2_O	
		A:B = 15:85 (*v*/*v*)	
	Wavelength	270 nm	
	Flow rate	0.45 mL/min	
(3) Column C Deactivated, extra dense bonding, double endcapped monomeric C18, high-purity silica, 3.0 mm × 250 mm, 5 µm particle size, and 10% carbon loading	Mobile phase Gradient elution	A: (90:10 MeOH-ACN) + 0.1% HOAc B: 0.1% HOAc in H_2_O	
		Time (min)	%A
		0	10
		2	10
		25	21
		28	25
		30	100
		35	10
	-	40	10
	Wavelength	210 nm	
	Flow rate	1.0 mL/min	

**Table 2:** Performance characteristics of column C during method validation and evaluation.

Compounds	Molecular Weight	Log P	Precision (% RSD)				Linear Range (mg/L)	Correlation Coefficient	LOD (mg/L)	LOQ (mg/L)
			Retention Time		Peak Area					
			Intraday	Interday	Intraday	Interday				
GA	170.12	0.70	1.02	1.25	0.69	0.87	2–20	0.9993	0.58	2.01
GC	306.27	1.49	0.89	1.07	0.77	0.92	2–15	0.9998	0.52	1.77
EGC	306.27	1.49	0.86	1.26	0.86	1.06	2–15	0.9994	0.64	2.09
C	290.27	1.80	1.05	1.28	0.82	0.98	2–20	0.9997	0.49	1.59
Caf.	194.19	−0.55	0.49	0.97	0.49	0.88	2–20	0.9998	0.23	0.67
EC	290.27	1.80	0.77	0.93	0.87	1.33	2–20	0.9995	0.68	2.18
EGCG	458.37	3.08	1.02	1.13	0.79	1.07	2–15	0.9994	0.58	1.86
GCG	458.37	3.08	0.65	0.88	0.95	1.17	2–20	0.9997	0.62	2.13
ECG	442.37	3.88	0.58	0.70	0.48	1.29	2–20	0.9997	0.33	1.07

**Table 3:** Mean recoveries of catechins and related compounds from the *Miang* extracts.

Compounds	*Miang* Extract-1		*Miang* Extract-2		*Miang* Extract-3	
	Recovery (%)	% RSD	Recovery (%)	% RSD	Recovery (%)	% RSD
GA	87–106	7	90–101	6	85–101	6
GC	85–101	6	85–98	4	85–97	5
EGC	89–101	5	85–97	7	88–102	6
C	88–102	5	93–102	5	95–105	5
Caf.	90–102	5	92–102	6	92–102	5
EC	86–96	4	89–98	5	91–102	6
EGCG	87–101	5	87–97	5	85–101	5
GCG	88–102	5	85–98	6	88–102	6
ECG	90–101	6	88–101	5	86–95	4

Mean value of three replicates for two concentrations (5LOQ and 10LOQ; *n* = 6).

**Table 4:** Amount of catechins and related compounds in *Miang* extracts.

Compounds	-	S1T1	S1T2	S1T3	S2T1	S2T2	S2T3	S3T1	S3T2	S3T3
GA	Mean	0.64	1.58	0.86	1.70	1.96	0.80	2.85	1.40	3.23
	%RSD	1.52	3.78	4.58	3.33	3.59	3.79	2.20	4.08	2.89
GC	Mean	3.52	3.47	4.23	2.61	1.64	1.35	3.62	1.98	1.18
	%RSD	2.21	1.75	2.15	3.67	3.40	1.52	2.82	4.29	4.58
EGC	Mean	8.90	19.41	9.51	4.75	1.04	1.76	4.71	4.07	4.41
	%RSD	3.38	1.09	1.47	1.49	4.24	3.45	1.95	1.48	4.35
C	Mean	45.05	18.19	16.75	8.16	5.44	6.21	8.49	8.16	9.46
	%RSD	1.10	2.04	3.58	2.63	4.25	3.76	2.64	2.63	2.93
Caf.	Mean	13.68	23.89	5.69	35.63	25.84	26.20	41.82	40.81	30.69
	%RSD	3.53	1.76	3.76	1.20	2.21	1.19	1.27	1.85	1.59
EC	Mean	2.91	13.04	15.62	1.14	0.82	3.23	7.78	3.55	10.48
	%RSD	4.67	4.61	4.41	4.14	3.08	3.96	3.96	3.93	4.79
EGCG	Mean	4.36	12.89	10.69	8.76	3.58	4.92	5.86	11.22	7.26
	%RSD	4.53	2.47	2.96	2.55	2.25	2.80	3.42	2.22	2.85
GCG	Mean	1.07	1.03	2.80	1.08	0.84	0.99	2.03	1.34	2.10
	%RSD	5.17	2.33	4.93	5.09	4.29	2.22	3.66	4.25	3.72
ECG	Mean	0.72	ND	1.62	ND	ND	0.39	0.52	0.81	0.86
	%RSD	2.76	-	5.12	-	-	3.74	3.83	4.44	4.28

Mean = average amount of each compound in mg/g of the *Miang* extract; mean value of three replicates and three injections for each replicate. ND = not detected (below LOD value). Extraction solvents 75% ethanol (S1), deionized water (S2), and 50% ethanol (S3) at 60, 70, and 80 °C (T1, T2, and T3), respectively).

## Data Availability

The original contributions to this study are included in this article. The data presented in this study are available upon request from the corresponding author.

## Sample Availability

Not available.
